# Ion-exchange recovery of cyanide from alkaline effluents: kinetic and equilibrium modelling under controlled alkaline conditions

**DOI:** 10.1007/s11356-026-38011-4

**Published:** 2026-07-10

**Authors:** Darlington Ashiegbu, Paballo Pilane, Olga Bazhko, Sherif Ishola Mustapha, Herman Potgieter

**Affiliations:** 1 School of Chemical and Metallurgical Engineering, University of the Witwatersrand, Johannesburg, South Africa; 2 Council for Mineral Technology (Mintek), Johannesburg, South Africa

**Keywords:** Ion exchange, Cyanide recovery, Pseudo-second-order kinetics, Alkaline adsorption, Purolite A500, Sustainable hydrometallurgy

## Abstract

In hydrometallurgical and electroplating processes, cyanide serves as a cornerstone reagent but poses substantial environmental and regulatory challenges due to its toxicity and persistence in effluents. This work establishes a rigorous calibration-first framework for cyanide recovery using strong-base ion-exchange resins under alkaline, process-relevant conditions. Matrix-matched cyanide ion selective electrode (ISE) calibrations were generated at pH values of 10–13 with validated slope, linearity, and stability criteria, enabling accurate speciation accounting and eliminating measurement uncertainty typical of high-alkalinity systems. Among the resins evaluated, Purolite A500-L, a type I strong-base macroporous anion-exchange resin based on a polystyrene-divinylbenzene matrix with quaternary ammonium functional groups and a total exchange capacity of approximately 1.15 eq L⁻^1^ (Cl⁻ form), demonstrated superior performance. Rapid cyanide uptake from an initial cyanide concentration of 20 mgL^−1^ occurred within the first 10–20 min, with > 90% removal at pH 11 and 1 g L⁻^1^ resin. Kinetic modelling showed excellent agreement with the pseudo-second-order model (*R*^2^ > 0.999) and close alignment between experimental and predicted capacities, indicating a chemisorption-controlled ion-exchange process. Langmuir isotherms provided the best fit to equilibrium data (*R*^2^ = 0.994–0.998), yielding high monolayer capacities (7.315–7.593 mg g⁻^1^) and increasing affinity constants (*K*_L_ = 0.129–0.247 L mg⁻^1^). Freundlich parameters supported favorable adsorption with moderate site heterogeneity. Overall, the study provides a decision-grade dataset for cyanide recovery modelling and demonstrates the necessity of analytical QA/QC in adsorption research. The methodology offers a robust foundation for designing sustainable, circular hydrometallurgical systems that recover cyanide while ensuring environmental compliance.

## Introduction

Cyanide remains indispensable in hydrometallurgical operations, particularly within carbon-in-pulp (CIP) and carbon-in-leach (CIL) circuits, where it functions as the principal lixiviant for the dissolution and recovery of precious metals such as gold and platinum (Fleming 1992; Van Deventer 1984; Altinkaya et al. 2020; Anderson 2016). Beyond gold extraction, cyanide has been employed in the treatment of platinum group metals and base metals, forming a diverse range of soluble metal cyanide complexes that contribute to the chemical complexity of process liquors and effluents (Vorob’ev-Desyatovskii et al. 2012; Snyders et al. 2012; Kim et al. 2024). Despite its operational efficacy, cyanide use has become increasingly scrutinized due to its acute toxicity, environmental persistence under alkaline conditions, and potential transformation into stable but hazardous secondary species such as thiocyanate (SCN⁻) and metal cyanide complexes (e.g., [Fe(CN)₆]^4^⁻, [Ni(CN)₄]^2^⁻) (Mirizadeh et al. 2014; Mekuto et al. 2016). Cyanide was selected in this study because it remains one of the most operationally important yet environmentally sensitive reagents in hydrometallurgical gold extraction and electroplating systems. Its strong metal-complexing ability makes it technically valuable, but its acute toxicity, environmental persistence under alkaline conditions, and ability to generate hydrogen cyanide under unfavorable pH conditions make its control and recovery scientifically and practically significant. Consequently, effluent compliance and resource circularity have become dual imperatives for the sustainable management of cyanide-bearing wastes.

In CIP/CIL systems, cyanide consumption frequently exceeds stoichiometric requirements due to reactions with base metals, oxygen, and sulfides, leading to significant cyanide losses (Marsden & House 2006). This results in tailings and process waters containing high levels of free and complexed CN⁻, which not only represent a considerable economic loss but also pose major regulatory challenges (Manaviparast et al. 2024). Similarly, electroplating and metal-finishing industries generate alkaline effluents containing cyanide and heavy metals, necessitating selective, cost-effective recovery processes (Zhao et al. 2025). Conventional destructive treatments such as alkaline chlorination, peroxide oxidation, and the INCO SO₂/Air process convert cyanide into less toxic species like cyanate (OCN⁻) or thiocyanate (SCN⁻), but simultaneously destroy the recoverable reagent value while producing secondary by-products that complicate waste management (Botz et al. 2001; Mediavilla et al. 2019; Kuyucak and Akcil 2013). In contrast, recovery-minded strategies like ion exchange (IX) and membrane-assisted regeneration offer the dual benefit of environmental compliance and resource reclamation, supporting the broader transition toward circular hydrometallurgy.

Ion-exchange processes have demonstrated considerable promise for cyanide recovery, leveraging functionalized polymeric resins capable of selectively adsorbing both free CN⁻ and weakly complexed cyanide species under alkaline conditions (Torre et al. 2006; Zhou et al. 2023). Such systems enable cyanide regeneration for reuse, reduce reagent consumption, and facilitate water recirculation, thus improving the sustainability of gold leaching and electroplating operations. Moreover, recent developments in membrane contactors and hybrid IX systems have achieved up to 70–100% regeneration of cyanide from metallurgical effluents, offering viable, scalable alternatives to oxidative destruction (Gonen et al. 2004; Hammer et al. 2023). Despite these advances, the recovery of uncomplexed cyanide as distinct from metal cyanide complexes remains insufficiently characterized. Most existing studies focus on synthetic or simplified systems, often neglecting the complex interplay between pH, ionic strength, and speciation that governs adsorption efficiency and selectivity.

A major limitation in the current literature is the lack of decision-grade data derived from analytically validated methods (Zheng et al. 2003). Cyanide quantification using ion selective electrodes (ISEs) is prone to some errors due to volatilization, complexation equilibria, and hydroxide interference (Gattrell et al. 2001), yet few studies implement robust calibration protocols or establish analytical quality assurance (QA) criteria. Inconsistent calibration procedures and unverified electrode slopes have led to biased results, undermining the reliability of reported adsorption capacities and kinetic constants. To the best of our knowledge, this is the first study to combine matrix-matched ISE calibration at pH ≥ 11 with systematic ion-exchange kinetic/isotherm evaluation for cyanide recovery; this allows decision-grade parameterization suitable for process design.

The present study addresses critical methodological gaps through a speciation-aware, calibration-first framework for evaluating the ion-exchange recovery of free cyanide under alkaline, process-relevant conditions. This integrated approach combines analytical precision, comprehensive batch experimentation, and mechanistic modelling within a unified methodology to deliver both scientific and operational value. A pH-matched ion selective electrode calibration protocol with defined acceptance criteria for slope linearity and stability was developed to ensure reproducible, traceable cyanide quantification in complex alkaline matrices. Building on this validated analytical foundation, the study systematically investigates the influence of pH, resin dosage, and initial cyanide concentration on sorption behavior, deriving kinetics, that delineate practical operating windows for cyanide recovery. The framework provides a rigorously validated calibration protocol, as well as a quality assured dataset that support process design and scale up. Collectively, these contributions advance understanding in cyanide science and promote methodological transparency in analytical hydrometallurgy. Additional studies on adsorption and ion-exchange-based removal systems have likewise shown that cyanide uptake depends strongly on solution chemistry, adsorbent functionality, and operating pH, further supporting the need for analytically rigorous, speciation-aware evaluation under controlled alkaline conditions (Pathania et al. 2016; Gupta et al. 2014, 2015). By coupling validated analytical measurements with predictive process modelling, this study transforms cyanide treatment from a compliance-driven necessity into a data-driven process, aligning with global sustainability imperatives and circular economy principles for responsible resource utilization.

## Materials and methods

### Reagents and materials

Sodium cyanide (NaCN, Sigma-Aldrich, ≥ 95%) and sodium hydroxide (NaOH, Sigma-Aldrich, ≥ 98%) were used as received. Distilled water was used for the preparation of all solutions. Three ion-exchange resins: Purolite FerrIX™ A33E, Purolite A500-L, and Purolite A100-L (Ecolab, USA) were supplied by Mintek, South Africa, and used as received.

All experiments were conducted using clean laboratory glassware, including volumetric flasks, beakers, pipettes, wash bottles, and low adsorption plasticware suitable for trace level cyanide handling. A calibrated pH meter equipped with standard buffer solutions was used for pH adjustment, while agitation during adsorption was achieved using a magnetic stirrer. Cyanide quantification was performed using a Mettler Toledo PerfectION™ combined cyanide ion selective electrode (ISE) connected to a precision millivolt (mV) meter. The electrode employed Ion Electrolyte B as the reference filling solution. Sample withdrawal during experiments was performed using 10-mL plastic syringes fitted with 0.25-μm microfilters to ensure particulate-free aliquots for analysis.

### Electrode preparation and ionic strength adjuster (ISA) solutions

The cyanide ion selective electrode was prepared and conditioned according to the manufacturer’s specifications and the *PerfectION™ Application Guidebook* (2010). The electrode was connected to an mV meter and filled with ion electrolyte B solution up to the filling hole, which was replenished regularly to maintain electrode stability and consistent reference pressure during measurements.

To ensure uniform background ionic strength in all standards and samples, ionic strength adjuster (ISA) solutions were prepared from NaOH and distilled water. A 10 M NaOH stock ISA was first prepared, followed by serial dilution to obtain 1 M and 0.1 M solutions. These ISAs were selected to correspond with experimental pH values of approximately 13, 12, and 11, respectively. The ISAs were used throughout calibration and sample measurement at a fixed ratio of 1 mL ISA per 100 mL of solution, ensuring consistent ionic strength and reproducible electrode response.

Prior to calibration, the electrode was subjected to a preliminary slope test to verify its performance. A 1000 mg L⁻^1^ NaCN stock solution was prepared, from which 100 mg L⁻^1^ and 10 mg L⁻^1^ working standards were derived via serial dilution. Each 100 mL standard was adjusted with 1 mL of 10 M NaOH ISA. The electrode slope which is defined as the change in millivolts per tenfold concentration change was determined to be –60 mV per decade, confirming optimal operation within the expected range of –54 to –60 mV/decade for standards measured at 20–25 °C (*PerfectION Guidebook*, Mettler-Toledo 2010).

To enhance electrode sensitivity at low cyanide levels, the ion selective electrode was soaked overnight in a 0.6 mg L⁻^1^ cyanide solution, prepared from serial dilution of a 100 mg L⁻^1^ NaCN standard with the corresponding ISA added in a 100:1 solution to ISA ratio. This conditioning step ensured consistent electrode response within its linear operating range, which typically begins at cyanide concentrations greater than 0.5 mg L⁻^1^.

### Batch adsorption studies

Batch adsorption experiments were carried out to evaluate the influence of key operating parameters on cyanide removal using ion-exchange resins. The preliminary set of experiments focused on resin screening to identify the most effective material among the three tested resins. Three identical stock solutions were prepared under controlled conditions (*C*₀ = 20 mg L⁻^1^, pH = 11, T = 25 °C, V = 100 mL, resin dose = 1 g, and ionic strength adjuster (ISA) dose = 1 mL). For each trial, 1 g of resin was introduced into the working solution, and the suspension was agitated at 150 rpm for 60 min. Samples were collected at 10-min intervals and analyzed for residual cyanide concentration. The resin demonstrating the highest cyanide removal efficiency was selected for subsequent kinetic and parametric studies. The initial screening dosage of 1 g per 100 mL was selected as a practical starting condition to ensure measurable uptake within the batch contact time, allow clear comparison among the three resins, and remain within a dosage range commonly used for exploratory batch ion-exchange screening.

A one-variable-at-a-time approach was employed to assess the effects of solution pH, resin dosage, and initial cyanide concentration, while keeping other parameters constant. The effect of pH was first investigated in the range of 10–13, as acidic conditions were deliberately avoided to prevent the evolution of toxic hydrogen cyanide (HCN) gas. For each pH experiment, 100 mL of distilled water was measured into a volumetric flask, followed by the addition of the required mass of solid NaCN. The mixture was transferred into a beaker, to which 1 mL of the designated ISA (10 M, 1 M, or 0.1 M NaOH) was added to achieve the target pH. The solution was stirred to ensure homogeneity before adding a pre-weighed amount of the selected resin. The suspension was then agitated at 150 rpm for 60 min, with samples withdrawn every 10 min for cyanide analysis using an ion selective electrode (ISE). Readings were recorded only after the meter stabilized.

The effect of resin dosage was examined next under the optimum pH condition (pH = 11), varying resin mass from 0.1 to 1 g per 100 mL of solution (equivalent to 1–10 g L⁻^1^), while maintaining *C₀* = 20 mg L⁻^1^, *T* = 25 °C, and an ISA dose of 1 mL (0.1 M NaOH). Finally, the influence of initial cyanide concentration was assessed at the optimized pH and resin dose by varying *C*₀ between 20 ^1^ and 100 mg L⁻^1^ under identical temperature, volume, and agitation conditions.

The removal efficiency (%) and the adsorption capacity (*Qₜ* or *Qₑ*), representing the amount of cyanide adsorbed per gram of resin, were determined using the mass balance relationship based on initial and equilibrium solute concentrations as shown in Eqs. 1 and 2, respectively.

1 $${\mathrm{R}\mathrm{e}\mathrm{m}\mathrm{o}\mathrm{v}\mathrm{a}\mathrm{l}\,\mathrm{e}\mathrm{f}\mathrm{f}\mathrm{i}\mathrm{c}\mathrm{i}\mathrm{e}\mathrm{n}\mathrm{c}\mathrm{y} (\%)}_{=} \frac{\left({C}_{o}-{C}_{t}\right)}{{C}_{o}}\times 100$$

2 $${Q}_{t=} \frac{({C}_{o}-{C}_{t})V}{m}$$

where *Q*_*t*_ is the adsorption capacity at time *t* (mg g⁻^1^), *C*_*o*_ is the initial cyanide concentration (mg L⁻^1^), *C*_*t*_ is the cyanide concentration at time *t* (mg L⁻^1^), *V* is the volume of the solution (L), and *m* is the mass of the resin used (g) (Ehsan et al. 2020).

### Adsorption kinetics

The adsorption kinetics provide vital insight into the rate and mechanism governing cyanide uptake by the selected ion-exchange resin. In this study, the adsorption kinetics were analyzed using the pseudo-first-order (PFO) and pseudo-second-order (PSO) models, which are widely applied to describe solute–solids interactions in aqueous systems.

The PFO model assumes that the rate of occupancy of adsorption sites is proportional to the number of unoccupied sites (Teweldebrihan et al. 2025). Its linearized form is expressed as follows:

3 $$\mathrm{ln}\left({Q}_{e}-{Q}_{t}\right)=ln{Q}_{e}-{k}_{1}t$$

where *Q*_*e*_ (mg g^−1^) and *Q*_*t*_ (mg g^−1^) represent the amounts of cyanide adsorbed at equilibrium and at time *t* (min), respectively, and *k*_1_ (min⁻^1^) is the PFO rate constant. A plot of *ln(Q*_*e*_* − Q*_*t*_*)* versus* t* was used to determine* k*_1_ and* Q*_*e*_ from the slope and intercept.

The PSO model, on the other hand, assumes that chemisorption involving valence forces through electron sharing or exchange governs the rate-controlling step (Mohamed Nasser et al. 2024). The linearized PSO equation is given by the following:

4 $$\frac{t}{{Q}_{t}}=\frac{1}{{k}_{2}{{Q}^{2}}_{e}}+\frac{t}{{Q}_{e}}$$

where *k*_2_ (g mg⁻^1^ min⁻^1^) is the PSO rate constant. A plot of *t/Q*_*t*_ against *t* yields a straight from which both *k*_2_ and *Q*_*e*_ can be obtained.

### Adsorption isotherms

Adsorption isotherm models provide critical insight into the equilibrium relationship between the adsorbed and residual concentrations of cyanide in solution (Behnamfard and Salarirad, 2009). They describe how solute molecules distribute between the liquid and solid phases under equilibrium conditions and are essential for the design and optimization of ion-exchange systems. In this study, the equilibrium data for cyanide adsorption onto the selected resin were analyzed using two widely applied models—Langmuir and Freundlich—to elucidate the adsorption mechanism and surface characteristics of the adsorbent.

The Langmuir isotherm assumes homogeneous adsorption sites with uniform energies and monolayer coverage of adsorbate molecules without lateral interactions between adjacent sites (Swenson and Stadie 2019). Its linearized form is represented as follows:

5 $$\frac{{C}_{e}}{{Q}_{e}}= \frac{1}{{K}_{L}{Q}_{\mathrm{m}\mathrm{a}\mathrm{x}}}+\frac{{C}_{e}}{{Q}_{\mathrm{m}\mathrm{a}\mathrm{x}}}$$

where *C*_*e*_ (mg L^−1^) is the equilibrium concentration of cyanide in solution, *Q*_*e*_ (mg g^−1^) is the amount adsorbed per unit mass of resin, *Q*_max_ (mg g⁻^1^) is the theoretical monolayer capacity, and *K*_L_ (L mg⁻^1^) is the Langmuir adsorption constant, which reflects the affinity of the active sites for the adsorbate (Simsek et al. 2015). From a plot of *C*_*e*_*/Q*_*e*_ versus *Ce*, *Q*max and *K*_L_ are obtained from the slope and intercept, respectively.

In contrast, the Freundlich isotherm accounts for heterogeneous surface energies and multilayer adsorption, assuming non-uniform distribution of active sites and variable adsorption affinities (Cojocaru et al. 2024). Its empirical linearized equation is given as follows:

6 $$\mathrm{l}\mathrm{o}\mathrm{g}{Q}_{e}=\mathrm{l}\mathrm{o}\mathrm{g}{K}_{F}+\frac{1}{n}\mathrm{l}\mathrm{o}\mathrm{g}{C}_{e}$$

where *K*_*F*_ ((mg g⁻^1^)(L mg⁻^1^)^1^⁄*ⁿ*) and *n* are Freundlich constants related to adsorption capacity and adsorption intensity, respectively. The magnitude of $\frac{1}{n}$ provides an indication of adsorption favorability: 0 <$\frac{1}{n}$  < 1 signifies a favorable process, while $\frac{1}{n}$ > 1 suggests cooperative adsorption behavior (Mustapha et al. 2025). The experimental data were fitted to both isotherm models to interpret the adsorption behavior of cyanide ions on the resin.

## Results and discussion

### Resin characteristics

Purolite FerrIX™ A33E is a macroporous polystyrenic resin cross-linked with divinylbenzene, engineered for arsenic and oxyanion removal. It consists of brown spherical beads with particle sizes between 300 and 1200 µm and a uniformity coefficient ≤ 1.7. The matrix is impregnated with nano-sized iron oxide particles that act as active adsorption sites for anionic species. The resin exhibits excellent mechanical stability and low pressure drop under typical flow rates (10–40 BV h⁻^1^). Operational pH is maintained between 4.5 and 8.5, and its temperature tolerance is ≤ 80 °C (Ecolab 2023).

Purolite A500-L is a type I strong-base anion-exchange resin with quaternary ammonium functional groups on a macroporous polystyrene–divinylbenzene backbone. It appears as spherical beads (0.6–0.85-mm average diameter) with a uniformity coefficient ≤ 1.7 and moisture content of 53–58%. This resin provides high total capacity (~ 1.15 eq L⁻^1^ in Cl⁻ form) and exceptional resistance to osmotic and mechanical stress. Its macroporous, isoporous architecture ensures efficient ion transport and high selectivity for negatively charged complexes, making it suitable for cyanide and metal cyanide complex recovery from process liquors (Lenntech 2023a, b).

Purolite A100-L is a macroporous weak-base anion-exchange resin featuring tertiary-amine functional groups on a polystyrenic matrix. It demonstrates high operating capacity for removing strong mineral acids and organic acids formed after decationization. The resin also exhibits excellent resistance to osmotic shock and mechanical breakage, with favorable elution characteristics requiring only ~ 125% of the stoichiometric NaOH equivalent during regeneration. Its porosity and chemical robustness make it well suited for removing organic contaminants and weakly complexed anions from wastewater (Lenntech 2023a, b).

### Calibration experiments

Matrix-matched calibration was performed to ensure accurate cyanide quantification under varying pH and ionic strength conditions. Separate calibration curves were constructed for each experimental pH (10, 11, 12, and 13) to maintain consistency between the standards and sample matrices. It is crucial that the standards used for calibration and the samples being measured possess equivalent ionic strengths, as differences in background ionic composition directly influence the electrode response and compromise the accuracy of interpolation from the calibration curve.

When the pH of the working solution is adjusted by varying the concentration or volume of NaOH, the ionic strength (I) of the solution also changes, as defined by Eq. (7):

7 $${\rm I}=0.5\sum {C}_{i}{Z}_{i}^{2}$$

The activity coefficient (*γ*), given in Eq. (8), represents the ratio between the effective concentration (activity) and the analytical concentration of ions in solution

8 $$\gamma =\frac{\mathrm{A}\mathrm{c}\mathrm{t}\mathrm{i}\mathrm{v}\mathrm{i}\mathrm{t}\mathrm{y}\,(\mathrm{i}\mathrm{n} \mathrm{c}\mathrm{o}\mathrm{n}\mathrm{c}\mathrm{e}\mathrm{n}\mathrm{t}\mathrm{r}\mathrm{a}\mathrm{t}\mathrm{i}\mathrm{o}\mathrm{n}\, \mathrm{u}\mathrm{n}\mathrm{i}\mathrm{t}\mathrm{s})}{\mathrm{C}\mathrm{o}\mathrm{n}\mathrm{c}\mathrm{e}\mathrm{n}\mathrm{t}\mathrm{r}\mathrm{a}\mathrm{t}\mathrm{i}\mathrm{o}\mathrm{n}}$$

The activity coefficient decreases with increasing ionic strength; thus, the discrepancy between the measured activity and the true concentration widens at higher ionic strengths. This means that at elevated NaOH concentrations, the electrode potential deviates from ideal Nernstian behavior, leading to potential errors in cyanide quantification. Therefore, if the ionic strength of the sample differs from that of the calibration standards, the resulting electrode readings become incompatible, producing erroneous interpolated concentrations. To eliminate this error, both the calibration standards and samples were adjusted to identical ionic strengths using a sodium hydroxide-based ionic strength adjuster (ISA) in a consistent 100:1 solution to ISA ratio, as recommended by the *PerfectION Guidebook* (Mettler-Toledo 2010). This procedure validates the importance of matrix-matched calibration rather than using a single, universal calibration curve across variable pH conditions. The concentrations of NaOH required to achieve the desired ionic strength were computed using the following relationships:

9 pH+pOH=14

10 $$pOH=-log{[OH]}^{-}$$

11 $$NaOH\to {Na}^{+}+{OH}^{-}$$

12 $${C}_{1}{V}_{1}={C}_{2}{V}_{2}$$

The corresponding NaOH ISA concentrations were determined as summarized in Table 1.

**Table 1:** NaOH ISA concentrations

pH	pOH	[OH⁻] (M)	*C*₂ = [NaOH](M)	*C*₁ = [ISA] (M)
13	1	0.1	0.1	10
12	2	0.01	0.01	1
11	3	0.001	0.001	0.1
10	4	0.0001	0.0001	0.001

**Fig. 1:**
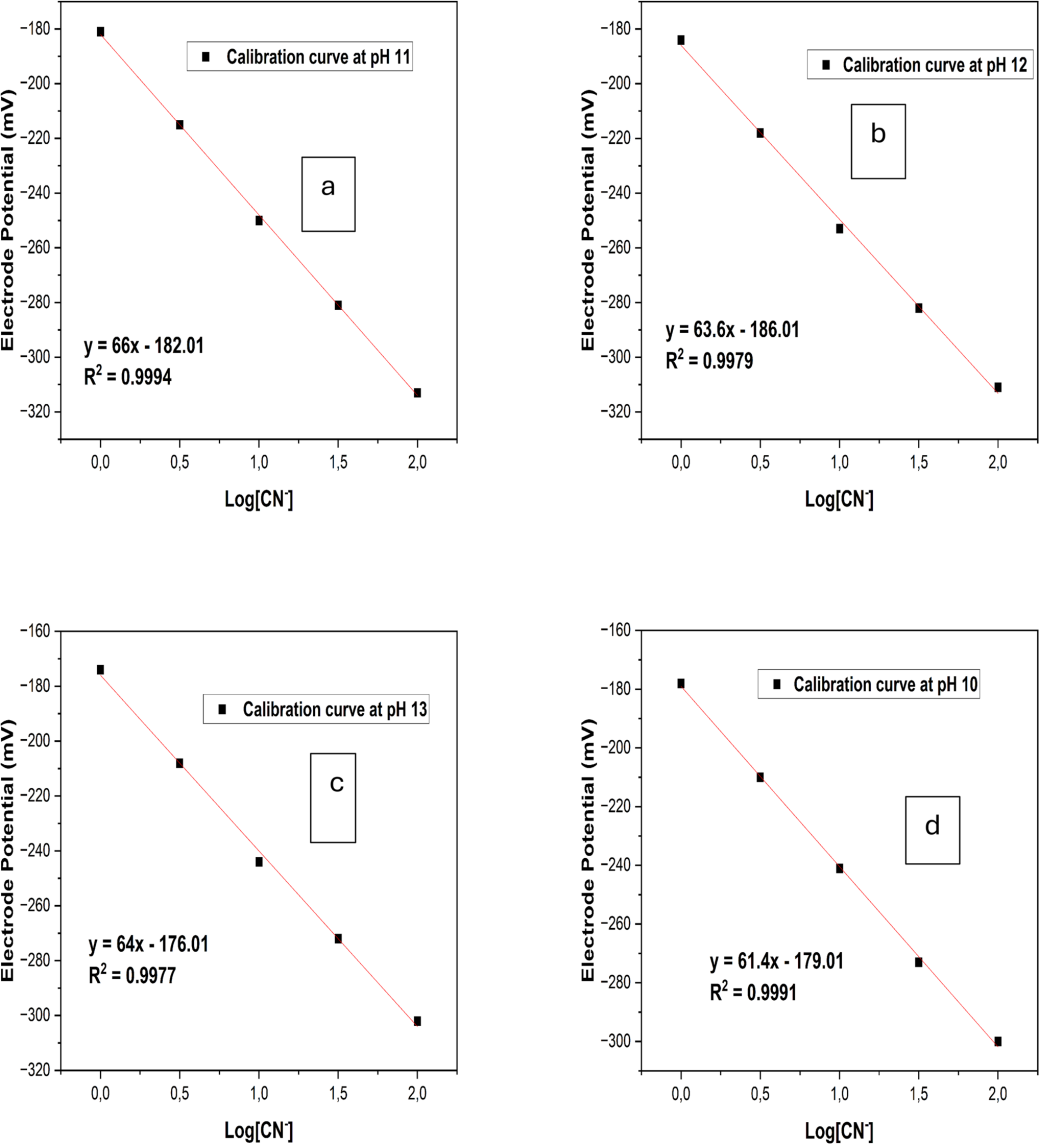
Calibration experiments at **a** pH 12, **b** pH 11, **c** pH 13, and **d** pH 10

All glassware was thoroughly cleaned with soap and tap water, rinsed with distilled water and dilute nitric acid (0.1 M), and dried before use. A 1000 mg L⁻^1^ NaCN stock solution was prepared using distilled water, from which five calibration standards were prepared by serial dilution over the concentration range 1–100 mg L⁻^1^. The calibration points used were 1, 3, 10, 30, and 100 mg L⁻^1^, consistent with the semi-log calibration plots shown in Fig. 1. Each 100 mL standard was adjusted with 1 mL of the appropriate NaOH ISA and stirred thoroughly to ensure homogeneity. The standards were analyzed sequentially from the lowest to highest concentration, with the electrode rinsed and blotted dry between readings. Electrode potential (E) was measured according to the Nernst equation (Eq. 13), which describes the logarithmic relationship between electrode potential and ionic activity:

13 $$E={E}^{0}-\frac{RT}{zF} \mathrm{l}\mathrm{n}Q$$

where *E* is the electrode potential (V), *E*⁰ is the standard electrode potential (V), *R* is the universal gas constant (8.314 J mol⁻^1^ K⁻^1^), *T* is the absolute temperature (K), *z* is the charge number of the ion, *F* is the Faraday constant (96 485 C mol⁻^1^), and *Q* is the reaction quotient representing the ratio of the activities (or effective concentrations) of the oxidized and reduced ionic species (Wendt & Kreysa 1999).

Calibration plots were constructed as semi-logarithmic graphs, where electrode potential (mV) was plotted against the logarithm of cyanide ion concentration, log[CN⁻], across different alkaline conditions (pH 10–13). The resulting calibration curves exhibited well-defined linear relationships, as shown in Fig. 1a–d, with regression equations of *E* =  *− *61.4*x –* 179.01 *(R*^2^ = 0.9991), *E* = *–*66*x –* 182.01 (*R*^2^ = 0.9994), *E* = *–*63.6*x –* 186.01 (*R*^2^ = 0.9979), and *E* = *–*64*x –* 176.01 (*R*^2^ = 0.9977) for pH 10, 11, 12, and 13, respectively. Each curve demonstrated excellent linearity, reproducibility, and Nernstian behavior, where a tenfold increase in cyanide concentration resulted in an approximately 61–66 mV potential change, closely matching the theoretical Nernstian slope for a monovalent anion at 25 °C. The near-identical slopes across all alkaline media confirm that electrode response was stable and independent of hydroxide ion interference, ensuring that the sensing mechanism was dominated by cyanide activity rather than competing ions.

Moreover, the high correlation coefficients highlight the precision and repeatability of the potential measurements, confirming both the robustness of the electrode and the reliability of the analytical protocol. The consistency in slope and intercept across pH 10–13 further validates the electrochemical stability of the ion selective membrane under strongly alkaline conditions. This pH-specific calibration strategy thus establishes a rigorous analytical foundation for cyanide quantification in speciation-aware studies. The near-Nernstian slopes and high linearity obtained in this study are consistent with expected cyanide ISE behavior under controlled ionic strength conditions, as reported in standard electroanalytical and cyanide ISE guidance literature (PerfectION Guidebook, Mettler-Toledo 2010; Wendt & Kreysa 1999; Zheng et al. 2003; Gattrell et al. 2001).

### Adsorption study

#### Resin selection

**Fig. 2:**
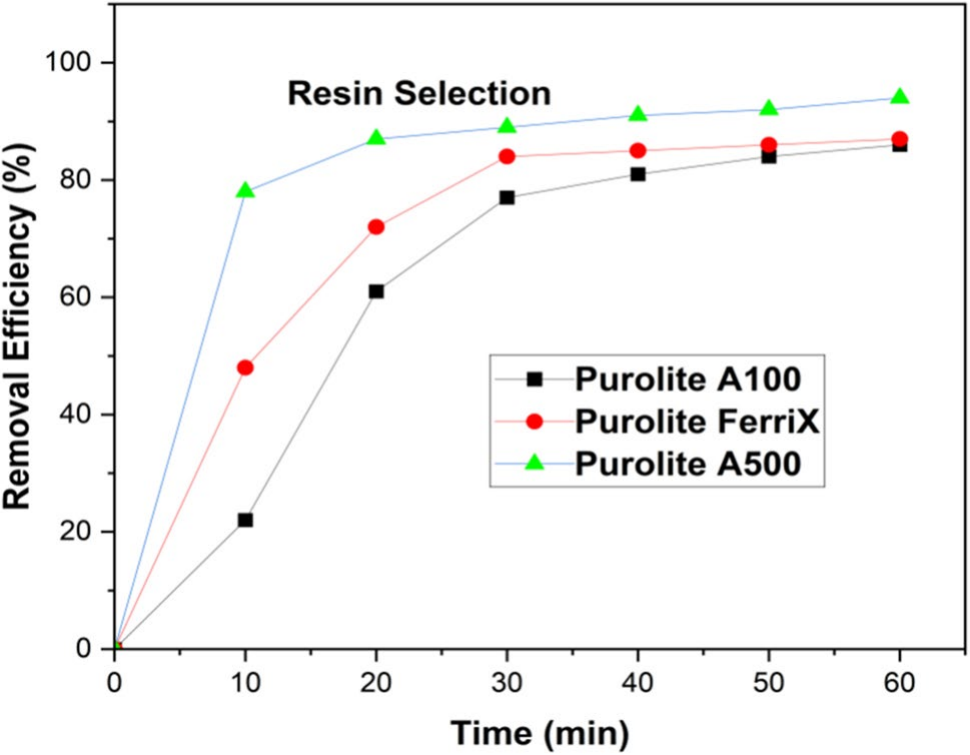
Comparative cyanide removal performance of three commercial ion-exchange resins (Purolite A500-L, FerrIX A33E, and Purolite A100-L) from alkaline cyanide solutions with an initial cyanide concentration of 20 mgL^−1^, at 25 °C, pH ≈ 11, resin dosage = 1 g per 100 mL, and agitation = 150 rpm

A preliminary screening experiment was conducted to identify the most effective ion-exchange resin for subsequent cyanide recovery studies. Three resins with distinct chemical functionalities—Purolite A100/2412, Purolite FerrIX™ A33E, and Purolite A500—were evaluated for their cyanide removal efficiency as a function of contact time, as presented in Fig. 2.

As shown in Fig. 2, the removal efficiency increased sharply within the first 10–20 min for all resins, suggesting rapid initial adsorption onto available exchange sites. Among the tested materials, Purolite A500 exhibited the highest removal efficiency, achieving approximately 80% removal within 10 min and reaching over 90% after 60 min, indicating fast ion-exchange kinetics and high affinity for cyanide species. Purolite FerrIX™ A33E demonstrated moderate performance, achieving about 75–85% removal at equilibrium, which is attributed to both surface complexation with Fe(III) sites and anion exchange within the polymeric matrix. In contrast, Purolite A100/2412 displayed a slower rate of cyanide uptake and a lower equilibrium removal (~ 85%), likely due to the partial protonation of tertiary amine sites in the weak-base fraction and limited availability of active quaternary ammonium groups under the alkaline experimental conditions. The overall trend of cyanide removal followed the order: Purolite A500 > Purolite FerrIX™ A33E > Purolite A100/2412.

This result shows that the strong base anion-exchange mechanism in Purolite A500 is the most effective for cyanide adsorption under the tested conditions, offering rapid attainment of equilibrium and high overall capacity. Consequently, Purolite A500 was selected as the preferred resin for subsequent batch adsorption, kinetic, and isotherm studies in this work. The superior performance of Purolite A500-L can be attributed to its strong-base quaternary ammonium functionality and macroporous structure, both of which favor rapid uptake of anionic species under strongly alkaline conditions. In contrast to weak-base resins, whose functionality depends on protonation state, type I strong-base resins retain exchange activity across a broader alkaline pH range. The macroporous architecture of A500-L also improves accessibility of active sites and facilitates mass transfer (Zhang et al. 2018).

#### Adsorption capacity of the selected resin (Purolite A500)

**Fig. 3:**
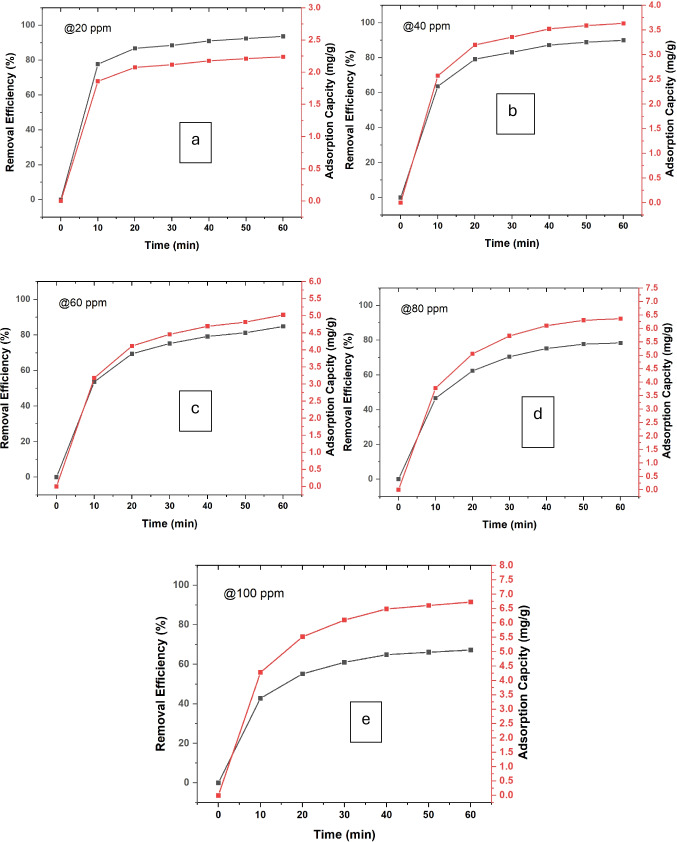
Effect of initial cyanide concentration (*C*_o_ = 20, 40, 60, 80, and 100 mgL^−1^) on adsorption capacity (*Qₑ*) and percentage removal by Purolite A500-L resin at pH ≈ 11, resin dosage = 1 g per 100 mL, temperature = 25 °C, and agitation speed = 150 rpm

The adsorption capacity (*Q*_*t*_ or *Q*_*e*_), representing the amount of cyanide adsorbed per gram of resin, was determined using the mass balance relationship based on initial and equilibrium solute conditions (Eq. 2). Figure 3 a–e illustrates the influence of initial cyanide concentration on the removal efficiency and adsorption capacity of the ion-exchange resin. At all concentrations, a rapid rise in both removal efficiency and adsorption capacity occurred within the first 10 min of contact time, followed by a gradual approach toward equilibrium after approximately 40–50 min. The sharp initial uptake is attributed to the large number of readily accessible active sites on the resin surface and the strong electrostatic attraction between cyanide anions and the positively charged quaternary ammonium functional groups (Zhang et al. 2018). As adsorption proceeded, these sites became progressively saturated, resulting in a reduced rate of uptake as equilibrium was approached.

At the lowest concentration (20 mg L⁻^1^), the resin achieved a removal efficiency of 94% and an equilibrium adsorption capacity of 2.24 mg g⁻^1^, confirming its strong affinity for cyanide ions under dilute conditions. When the initial cyanide concentration was increased to 40 mg L⁻^1^, a slight reduction in percentage removal (90%) was observed, accompanied by an increase in adsorption capacity to 3.63 mg g⁻^1^, reflecting the higher concentration gradient that enhances the mass transfer of cyanide ions to the resin surface. At an intermediate concentration of 60 mg L⁻^1^, the adsorption capacity further increased to 5.02 mg g⁻^1^, with a corresponding removal efficiency of approximately 85%, indicating that while the resin remains highly effective, partial site occupation and diffusion constraints begin to manifest. Similarly, at 80 mg L⁻^1^, the equilibrium capacity reached 6.36 mg g⁻^1^, whereas removal efficiency declined to 78%, suggesting a higher driving force for mass transfer but progressive site saturation. At the highest concentration tested (100 mg L⁻^1^), the adsorption capacity peaked at 6.72 mg g⁻^1^, while the removal efficiency decreased to 67%, implying that a fraction of cyanide ions remained unadsorbed due to limited availability of exchange sites and intra-particle diffusion resistance.

This behavior typifies the inverse relationship between removal efficiency and initial solute concentration frequently reported for ion-exchange and adsorption systems. At low concentrations, active sites are abundant relative to the number of cyanide ions, facilitating near complete removal. As the concentration increases, the adsorbate to adsorbent ratio rises sharply, leading to competition among cyanide ions for active sites and consequently reduced fractional removal (Liu et al. 2021; Alonso-González et al. 2013; Huang et al. 2025). The consistent increase in adsorption capacity with higher cyanide loading demonstrates that the resin maintains strong binding performance and high selectivity even under elevated cyanide concentrations.

#### Effect of pH on cyanide uptake

**Fig. 4:**
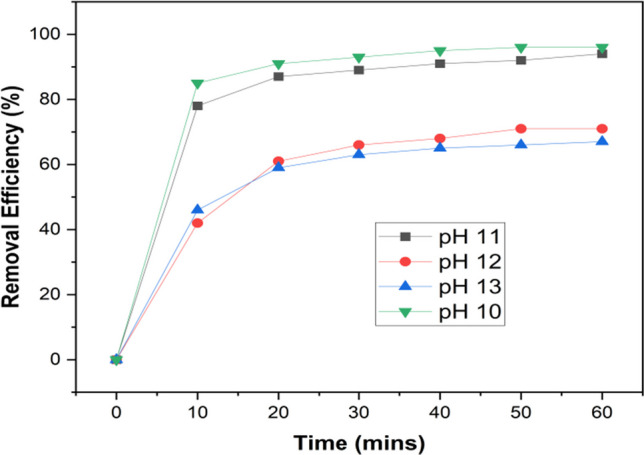
Effect of solution pH on cyanide removal efficiency by Purolite A500-L resin (initial cyanide concentration = 20 mg L⁻.^1^, resin dosage = 1 g per 100 mL, temperature = 25 °C, and agitation speed = 150 rpm)

Batch adsorption tests were conducted at *T* = 25 °C, *V* = 100 mL, (m_resin) = 1.0 g, and 150 rpm using Purolite A500 (*C*₀ = 20 mg L⁻^1^). The equilibrium pH was adjusted using 10 M NaOH to target pH ≈ 10, 11, 12, and 13. The time-dependent removal efficiencies for each pH are shown in Fig. 4.

The pH influence is evident. At pH ≈ 11, adsorption proceeds rapidly, reaching ≈ 80% removal within 10–20 min and attaining ≈ 94% by 60 min. A similar trend is observed at pH ≈ 10, which reaches ≈ 85% in 10–20 min and approaches ≈ 96% removal at 60 min. In contrast, removals at pH ≈ 12 and pH ≈ 13 are noticeably slower and plateau significantly lower, at only ≈ 70% after 50–60 min.

Although all sites on the type I quaternary ammonium resin remain permanently charged, the reduced performance at pH ≥ 12 is not due to loss of functional groups (Kassar et al. 2022). Instead, the decline originates from increased ionic strength and strong OH⁻ competition for exchange sites, which depress cyanide activity in solution and weaken Donnan partitioning (Simsek et al. 2015). The excess hydroxide ions restrict cyanide transport into the resin phase (Vincent et al. 2015), ultimately suppressing CN⁻ uptake (Al-Sakaji et al. 2023). Thus, any expected benefit of greater CN⁻ availability at extreme alkalinity is offset by these competing electrolyte effects (Chen et al. 2020).

While uptake efficiencies at pH ≈ 10 and pH ≈ 11 were both high (> 94%), pH 11 was selected for full optimization because it provides a stronger margin of cyanide speciation stability. With pKa(HCN) ≈ 9.2 (25 °C), operation near pH 11 ensures that cyanide exists overwhelmingly as CN⁻, whereas at pH 10, a small but non-zero fraction remains as molecular HCN. This aligns with standard industrial practice in CIP/CIL circuits, where pH values of 10.5–11 are adopted to suppress volatilization risks. The analytical measurements at pH 11 were also highly reproducible across experimental runs and showed stable ISE calibration behavior. Moreover, the slight difference between equilibrium removals at pH 10 (≈ 96%) and pH 11 (≈ 94%) falls within typical analytical variability, i.e., both values represent near-complete uptake. Thus, pH 11 offers an optimal balance between removal performance, analytical robustness, and safe cyanide speciation conditions. Lower pH conditions were intentionally excluded from this study because the objective was to evaluate free cyanide recovery under process-relevant alkaline conditions, while also avoiding the risk of hydrogen cyanide evolution associated with acidic or near-neutral media.

#### Effect of resin dose on cyanide removal efficiency

**Fig. 5:**
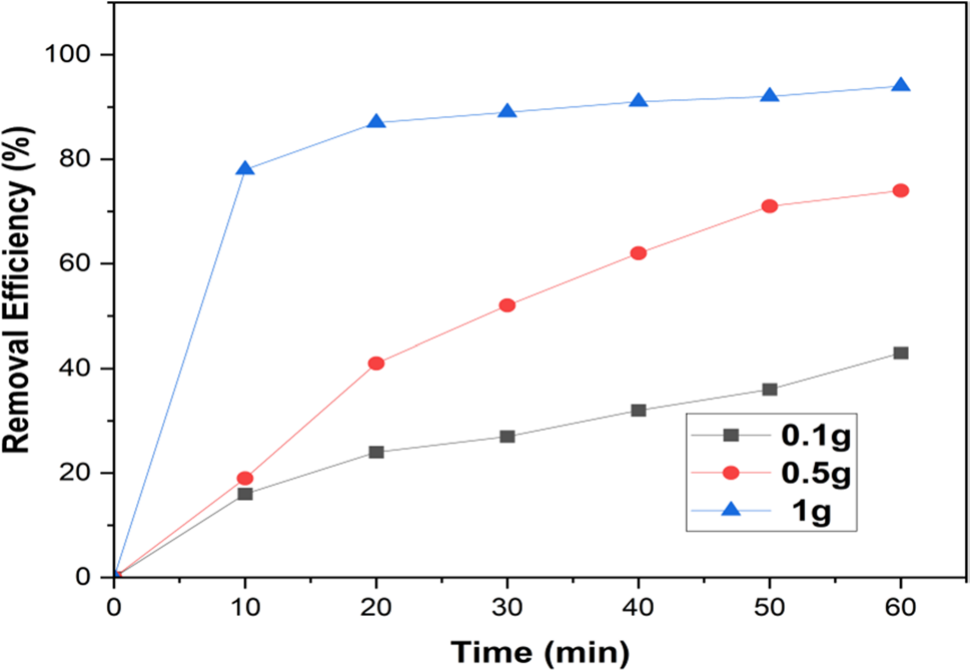
Effect of resin dosage on cyanide removal efficiency and adsorption capacity of Purolite A500-L resin (initial cyanide concentration = 20 mg L⁻.^1^, pH ≈ 11, temperature = 25 °C, and agitation speed = 150 rpm)

The influence of resin dosage on cyanide removal efficiency was evaluated using the SBA resin under the same optimized conditions. The resin mass was varied at 0.1 g, 0.5 g, and 1.0 g, and the results are presented in Fig. 5. A pronounced increase in removal efficiency was observed with increasing resin dosage. At the lowest dosage (0.1 g), cyanide removal reached only about 43% after 60 min, indicating limited availability of active exchange sites relative to the solution concentration. Increasing the resin mass to 0.5 g substantially improved removal, achieving ≈ 74% efficiency, while further increasing it to 1.0 g resulted in rapid and nearly complete removal (~ 87%) within the first 20 min. The higher resin loading provides a greater number of accessible quaternary ammonium sites and reduces interparticle diffusion resistance, leading to enhanced adsorption kinetics and faster attainment of equilibrium (Zhang et al. 2023; Meng et al. 2025).

These results confirm that adsorption performance is strongly dependent on resin mass (Wang et al. 2017), with 1.0 g of Purolite A500 offering the most effective balance between rapid kinetics and high overall cyanide removal.

### Adsorption kinetics

**Fig. 6:**
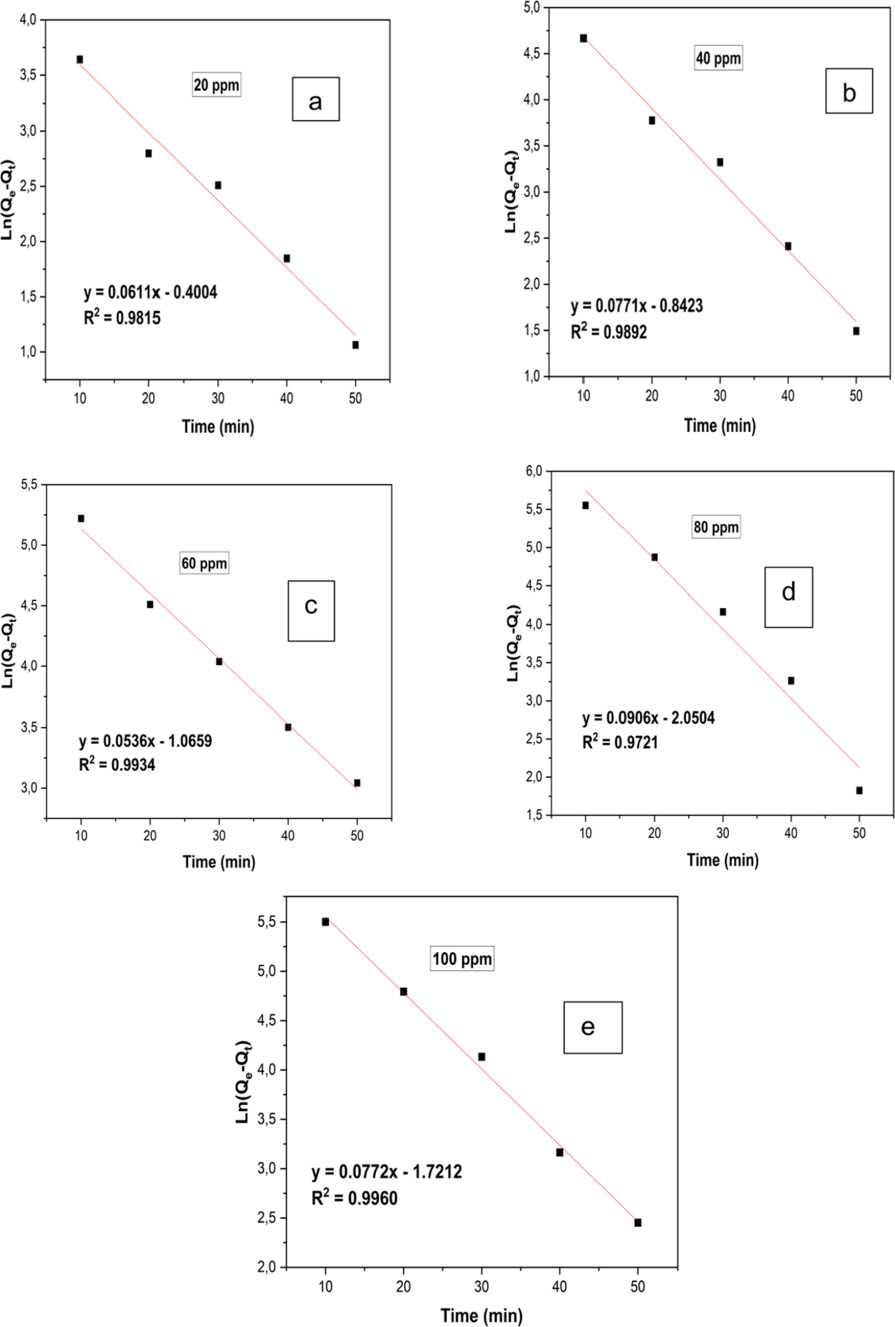
Pseudo-first-order plots at different concentrations. **a** 20 ppm. **b** 40 ppm. **c** 60 ppm. **d** 80 ppm. **e** 100 ppm

**Fig. 7:**
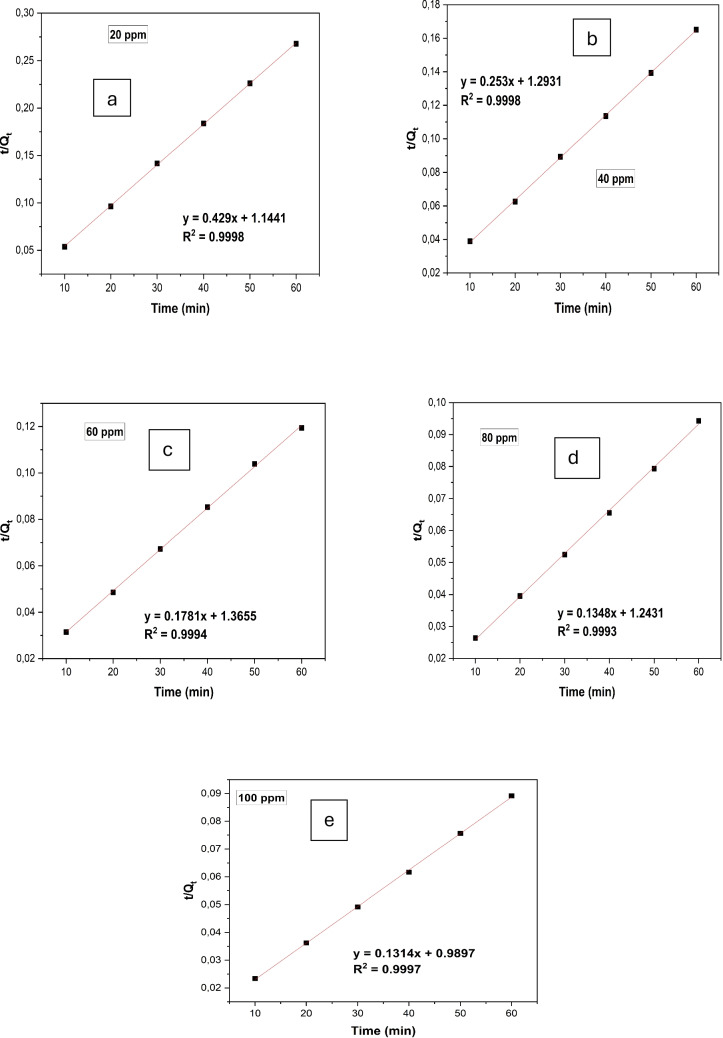
Pseudo-second-order plots at different initial concentrations. **a** 20 ppm. **b** 40 ppm. **c** 60 ppm. **d** 80 ppm. **e** 100 ppm

The adsorption kinetics describe the rate at which cyanide ions are transferred from the aqueous phase to the active sites of the ion-exchange resin and help elucidate the nature of the sorption mechanism. To interpret the experimental data, two kinetic models (the pseudo-first-order (PFO) and pseudo-second-order (PSO) models) were applied. These models were selected because they adequately capture the behavior of liquid–solid adsorption processes governed by either physical or chemical interactions. The plots are presented in Fig. 6a–e for PFO and Fig. 7a–e for PSO.

The linear plots of ln(*Qₑ − Qₜ*) versus time (*t*) at different cyanide concentrations for the PFO model exhibited strong linearity with correlation coefficients (*R*^2^) ranging from 0.9721 to 0.9960, indicating that the model provides a reasonable empirical description of the early adsorption phase. The corresponding rate constants (*k*₁) varied between 0.0536 and 0.0906 min⁻^1^ (Table 2), reflecting relatively fast initial uptake that diminished as equilibrium was approached. However, despite the high *R*^2^ values, minor discrepancies between the calculated and experimental equilibrium adsorption capacities (*Qₑ*) suggest that the PFO model only partially captures the true adsorption mechanism, likely due to its limited treatment of surface heterogeneity and active-site saturation dynamics.

**Table 2:** Kinetic model parameters for cyanide adsorption onto Purolite A500-L resin under controlled alkaline conditions

Concentration (ppm)	Pseudo-first-order			Pseudo-second-order		
	*Qe* (mg/g)	*K*_1_ (min^−1^)	*R*^2^	*Qe* (mg/g)	*k*_2_ (min^−1^)	*R*^2^
20	0.670	0.0611	0.9815	2.331	0.3750	0.9998
40	2.322	0.0771	0.9892	3.953	0.1957	0.9998
60	2.903	0.0536	0.9934	5.615	0.1304	0.9994
80	7.771	0.0906	0.9721	7.418	0.1084	0.9993
100	5.591	0.0772	0.9960	7.610	0.1328	0.9997

In contrast, the PSO model provided an excellent fit across all cyanide concentrations, as confirmed by the near perfect linearity (*R*^2^ > 0.999) in the plots of *t/Qₜ* versus* t*. The PSO rate constants (*k*₂) were found to decrease systematically from 0.3750 g mg⁻^1^ min⁻^1^ at 20 ppm to 0.1328 g mg⁻^1^ min⁻^1^ at 100 ppm, reflecting the influence of solute loading and available binding sites. Moreover, the *Qₑ* values predicted by the PSO model closely matched the experimental data, validating that cyanide adsorption follows a second-order rate law. This outcome implies that chemisorption governs the overall process, dominated by valence-controlled ion exchange and specific electrostatic interactions between cyanide anions (CN⁻) and the quaternary ammonium functional groups of the resin (Chen et al. 2019; Simsek et al. 2015; Zhang et al. 2018).

The higher correlation coefficients and smaller deviations in *Qₑ* predicted by the PSO model compared with the PFO model confirm that the adsorption process proceeds primarily through chemical ion exchange rather than physical diffusion. The initial stage of adsorption is characterized by rapid external mass transfer driven by a strong concentration gradient between the bulk solution and the resin surface. This two-phase kinetic pattern, comprising an initial film-diffusion phase followed by a chemisorption-controlled regime, is consistent with adsorption behaviors reported for similar anionic species on polymeric and hybrid ion-exchange systems (Liu et al. 2021; Alonso-González et al. 2013; Huang et al. 2025). The variations in *k*₂ across concentrations reveal the effect of cyanide loading on active site accessibility: at lower concentrations, abundant exchange sites facilitate rapid binding, whereas at higher loadings, competitive occupancy and diffusion constraints slightly reduce the apparent rate.

### Adsorption isotherms

Adsorption isotherms are essential for understanding the equilibrium relationship between the concentration of solute in the liquid phase and the amount adsorbed on the solid surface. They provide insight into the interaction between adsorbate and adsorbent as well as the distribution of active sites on the adsorbent surface. The equilibrium data for cyanide adsorption onto the ion-exchange resin were analyzed using the Langmuir and Freundlich models to describe the adsorption behavior of the resin.

**Fig. 8:**
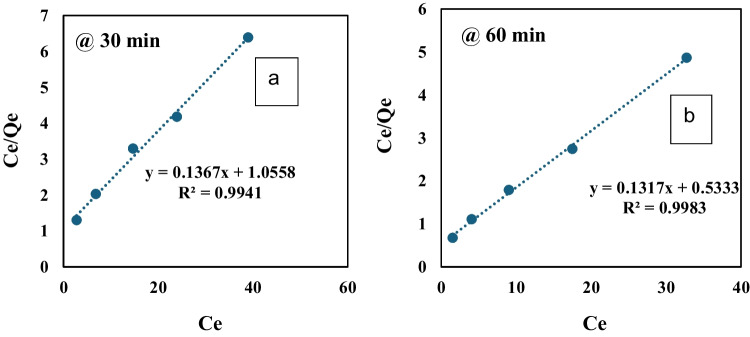
Langmuir isotherms at different contact times. **a** 30 min. **b** 60 min

**Fig. 9:**
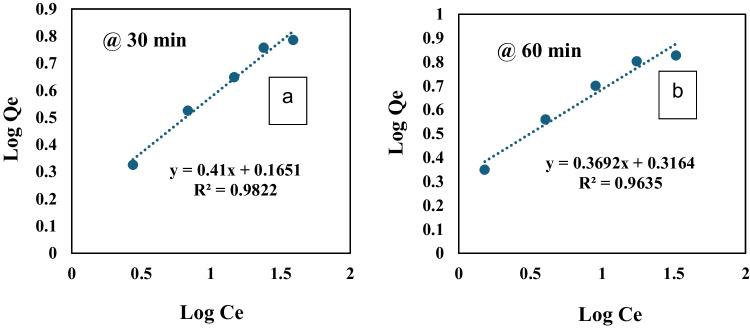
Freundlich isotherms at different contact times. **a** 30 min. **b** 60 min

The Langmuir plots presented in Fig. 8 yielded strong linear relationships with correlation coefficients (*R*2) of 0.9941 and 0.9983 at 30 and 60 min (Table 3^), respectively, outperforming the Freundlich fits (^*R*2 = 0.9822 and 0.9635) (shown in Fig. 9). This superior agreement indicates that cyanide adsorption onto the ion-exchange resin predominantly follows monolayer coverage on a homogeneous surface under the tested conditions. The high degree of linearity and the close correspondence between experimental and calculated adsorption values suggest that the resin possesses energetically equivalent active sites, each capable of binding a single cyanide ion until saturation is reached.

**Table 3:** Equilibrium isotherm parameters for cyanide adsorption onto Purolite A500-L resin under controlled alkaline conditions

	Freundlich			Langmuir		
Time (min)	*K*_*f*_ (mg/g)	${~}^{1}\!\left/ \!{~}_{{\boldsymbol{n}}}\right.$	*R*^2^	*Q*_max_ (mg/g)	*K*_L_ (L/mg)	*R*^2^
30	1.463	0.410	0.9822	7.315	0.129	0.9941
60	2.072	0.369	0.9635	7.593	0.247	0.9983

The maximum monolayer adsorption capacity *Q*_max_ obtained from the Langmuir model increased from 7.315 to 7.593 mg·g⁻1 as contact time extended from 30 to 60 min, indicating progressive occupation of available binding sites as the system approached equilibrium. Simultaneously, the Langmuir affinity constant *K*_L_ rose from 0.129 to 0.247 L·mg⁻1, suggesting stronger sorbate–sorbent interactions with increasing residence time and a more complete approach to equilibrium. This behavior is consistent with the gradual enhancement of ion-exchange interactions between cyanide anions and the quaternary ammonium functional groups within the resin matrix, which facilitate site-specific binding and controlled surface saturation.

Complementary fitting to the Freundlich model revealed additional insights into surface heterogeneity. The Freundlich constants showed that *K*_F_ increased from 1.463 to 2.072 mg·g⁻1, while ${~}^{1}\!\left/ \!{~}_{n}\right.$ decreased from 0.410 to 0.369, corresponding to n=2.44–2.71. Since 1<n<10, adsorption is considered favorable, and the decreasing ${~}^{1}\!\left/ \!{~}_{n}\right.$ values with time reflect an increasing dominance of higher affinity sites as equilibrium progressed. These results confirm that although the resin surface exhibits slight heterogeneity, adsorption remains efficient and favorable over the studied concentration range. The results imply that cyanide uptake proceeds mainly through chemisorption and ion-exchange mechanisms on a uniform active surface, supported by the high binding affinities and reproducibility across the studied times. These parameters provide a solid foundation for preliminary design and scale-up of ion-exchange systems, particularly in cyanide recovery and effluent treatment applications, where rapid equilibrium attainment and strong site affinity are desirable.

### Comparison with literature, post-use resin management, and practical implications

The present results extend beyond kinetic and equilibrium fitting by clarifying the practical behavior of different resin classes under strong alkaline cyanide conditions. The superior performance of Purolite A500-L is consistent with the general preference in cyanide recovery systems for strong-base anion-exchange resins carrying quaternary ammonium functional groups, particularly where free cyanide is present in alkaline solution, and resin performance must be maintained at pH ≥ 11 (Van Deventer 2014; Marston & Gisch 2010; Simsek et al. 2015). In the present study, A500-L showed the highest uptake rate and removal efficiency under the tested conditions, achieving rapid cyanide removal within the first 10–20 min at an initial cyanide concentration of 20 mg L⁻^1^, pH 11, and 1 g resin per 100 mL. This behavior agrees with the expected advantage of a macroporous strong-base resin, with high accessibility of exchange sites and effective uptake of anionic species under alkaline conditions. By contrast, the weaker performance of A100-L can be attributed to its weak-base tertiary amine functionality, which is less favorable at high pH where protonation is limited. FerrIX A33E also performed less effectively under the conditions used in this study, which is reasonable given that this resin is primarily designed for oxyanion removal and is typically operated in a lower pH window than that used here (Bazhko & Van Deventer 2022; Yang et al. 2015; Fernando et al. 2005; Marino & Kreuer 2015; Rajasingam et al. 2006; Kaušpėdienė et al. 2016). These differences highlight an important practical point: resin selection for cyanide recovery cannot be based on nominal exchange capacity alone, but must consider resin functionality, pH stability, and compatibility with the dominant cyanide species in solution.

From a practical standpoint, post-use resin management is a critical part of any recovery process. After cyanide loading, the resin should not be viewed as spent waste but as a recoverable process medium. In a practical system, the loaded resin would be separated from solution, washed under controlled alkaline conditions, and regenerated in a closed alkaline circuit to avoid hydrogen cyanide formation. The cyanide-rich regenerant could then be returned to the process where reuse is feasible, thereby supporting circular reagent management and reducing both reagent loss and wastewater burden. Resin recovery and regeneration would also need to be evaluated in terms of mechanical stability, retention of exchange capacity, and the effect of repeated loading-regeneration cycles. Although regeneration was outside the scope of the present batch study, the current results provide a suitable analytical and performance basis for such future work.

In terms of future practical application, Purolite A500-L appears to be the most promising of the three resins tested for alkaline free-cyanide recovery. Its strong base character, rapid uptake, and high removal efficiency at pH 11 suggest that it is better suited to process streams where cyanide is maintained in free ionic form under alkaline conditions, such as selected hydrometallurgical and electroplating liquors. The current batch data therefore identify A500-L as the most suitable candidate for further scale-up studies. Future work should focus on dynamic column testing, regeneration efficiency, competitive ion effects, breakthrough behavior, and validation with real industrial effluents. Such studies are necessary before full process implementation, but the present work already establishes a clear technical basis for selecting A500-L as the lead resin for further development.

## Conclusion

This study established a calibration-first and speciation-aware framework for evaluating free cyanide recovery by ion exchange under alkaline conditions relevant to hydrometallurgical and electroplating effluents. Matrix-matched cyanide ion selective electrode calibration provided reliable analytical control across pH 10–13, reducing uncertainty associated with high-alkalinity measurements. Among the resins tested, Purolite A500-L showed the highest removal efficiency and fastest uptake, consistent with its strong-base quaternary ammonium functionality and macroporous structure. Kinetic analysis showed that cyanide uptake was best described by the pseudo-second-order model, while equilibrium data were more closely represented by the Langmuir isotherm. The study therefore provides a robust analytical and modelling basis for cyanide recovery under controlled alkaline conditions, while also identifying Purolite A500-L as the most promising resin for future regeneration, column, and real effluent studies.

## Data Availability

Data to be made available upon request.
